# Exploring the role of standards-based descriptors in promoting translation learners’ metacognitive strategy use and translation performance

**DOI:** 10.3389/fpsyg.2025.1631662

**Published:** 2025-08-19

**Authors:** Huan Mei, Yingying Chen

**Affiliations:** ^1^School of Foreign Languages, Central China Normal University, Wuhan, China; ^2^School of Foreign Studies, Nanjing Forestry University, Nanjing, China; ^3^School of English Studies, Shanghai International Studies University, Shanghai, China

**Keywords:** China’s standards of English language ability, metacognitive strategy use, metacognitive support, standards-based descriptors, translation performance

## Abstract

This study explores the role of China’s Standards of English Language Ability (CSE) descriptors as metacognitive support in promoting translation learners’ metacognitive strategy use and translation performance. Forty students from two parallel classes participated, with the experimental group (*n* = 20) provided with CSE metacognitive descriptors during the translation course while the control group (*n* = 20) was not. Adopting a pretest-posttest design, qualitative analysis of students’ translation processes and products were conducted to investigate students’ use of metacognitive strategies. Quantitative assessment of students’ translation output was conducted to investigate their translation performance. Moreover, qualitative data from journal entries were analyzed to explore students’ perceptions of the metacognitive support. Results showed that CSE descriptors had an observable impact on translation learners’ metacognitive strategy use. However, the presence of metacognitive support did not significantly affect students’ overall translation performance. The study concludes that standards-based descriptors can be useful pedagogical tools for metacognitive translator training.

## 1 Introduction

Defined as “cognition about cognitive phenomena” (Flavell, 1979, p. 906), metacognition generally includes knowledge of cognition and regulation of cognition (Brown, 1987). Knowledge of cognition is concerned with what learners know about themselves and others as cognitive processors (Schraw and Sperling Dennison, 1994). Regulation of cognition, considered as the core and essence of metacognition (Reder and Schunn, 1996), involves the self-regulatory strategies learners employ to manage their learning processes, such as planning, monitoring and evaluating (Schraw, 1998).

In recent decades, there has been growing scholarly interest in metacognition and its development within educational research, particularly in metacognitive training (Braad et al., 2022; De Backer et al., 2015; Hacker et al., 2009; Kim and Lim, 2018). However, research on metacognitive training remains scarce in translation education, with only a few studies exploring this area (e.g., Fernández and Zabalbeascoa, 2012a; Hu et al., 2021; Li and Yuan, 2022). It is important to note that translation is inherently a cognitive process involving various cognitive subprocesses such as reading, text comprehension, semantic transfer and writing (Shreve, 2009; Shreve and Lacruz, 2017). Successful translation requires effective regulation of these cognitive processes, an ability intrinsically linked to metacognition (Pietrzak, 2022). In this context, metacognitive training plays a crucial role in translation education.

The importance of metacognition in translation is further evidenced by its inclusion in the well-established language standards such as the Common European Framework of Reference for Languages (CEFR; Council of Europe, 2018) and its Chinese counterpart, China’s Standards of English Language Ability (CSE; National Education Examinations Authority, 2018). Both standards incorporate strategic competence scales that explicitly feature metacognitive translation descriptors. Specifically, CEFR primarily focuses on while-translating phase with descriptors centered on the strategies to address specific translation problems (e.g., explain a new concept, simplify a text), by contrast, CSE covers all the three translation phases with different metacognitive focuses: pre-translating (planning), while-translating (execution & monitoring) and post-translating (evaluation & revision). To elaborate, “planning” involves making preparations for the entire translation process based on the translation purpose; “execution & monitoring” are concerned with utilizing translation techniques (e.g., addition, omission, conversion) to solve translation problems; “evaluation & revision” entail appraising the translation processes and products, and compensating for translation deficiencies through revision and cross-checking (Feng and Yan, 2020). Despite their availability, however, the metacognitive descriptors of these standards have rarely been utilized for instructional purposes. For instance, the CSE descriptors have primarily been used for assessment purposes in previous studies (e.g., Mei and Chen, 2022), while their potential for metacognitive training remains largely unexplored. This represents a major gap that this study aims to address.

## 2 Literature review

### 2.1 Metacognitive training and translation education

In the field of metacognition, a distinction has been made between metacognitive knowledge and metacognitive strategies (Brown et al., 1983). On the one hand, metacognitive knowledge refers to the individual’s declarative knowledge about the person, task, and strategies (Flavell and Wellman, 1977). Specifically, person knowledge refers to knowledge of one’s cognitive processes; Task knowledge concerns the purpose, nature, and demands of learning tasks; Strategy knowledge is knowledge of the strategies that can be used to achieve the cognitive goals of learning tasks (Lee and Mak, 2018). On the other hand, metacognitive strategies, which are comparable to metacognitive skills, denote the self-regulation activities (e.g., planning, monitoring, evaluating) that take place in the learning and problem-solving process (Bannert and Mengelkamp, 2013). A third category, named metacognitive experiences, has also been added (Flavell, 1979) and has gained increasing popularity in recent years (Sun and Zhang, 2023; Sun et al., 2024). In the present study, the focus is on students’ use of metacognitive strategies. Conceptualizations of metacognitive strategies vary across studies, encompassing components such as goal setting, orientation, strategy selection, planning, monitoring strategy execution, checking, evaluation, and debugging (Bannert and Mengelkamp, 2013; Hu et al., 2021; Schraw and Sperling Dennison, 1994). Nevertheless, three essential strategies have been included in all accounts: planning, monitoring, and evaluation (Jacobs and Paris, 1987; Schraw, 1998). The present study adopts this established conceptualization, which is well aligned with the metacognitive translation components specified in the CSE (i.e., planning, execution & monitoring, evaluation & revision).

Metacognitive training is about “the provision of explicit guidance to facilitate and support students’ reflection, monitoring, and evaluation of the metacognitive processes so that students are aware of their deployment of metacognitive knowledge and strategies” (Lee and Mak, 2018, p. 1089). The training typically involves both direct and indirect measures. The direct method requires instructors to explicitly explain metacognitive knowledge and strategies while demonstrating their application (Bannert and Mengelkamp, 2013). The indirect method integrates metacognitive support (e.g., scaffolds or prompts) into the learning environment to guide students in performing specific metacognitive activities (Bannert, 2006; Bannert et al., 2009). Previous research has demonstrated that metacognitive training can increase students’ metacognitive awareness, promote metacognitive strategy use and ultimately contribute to better learning performance (Künsting et al., 2013; Urban et al., 2023; Zumbach et al., 2020).

Metacognition has been consistently recognized as a key component of translation competence (PACTE, 2005; Yang and Li, 2021) with numerous studies stressing the significance of metacognitive training in translation education (Echeverri, 2015; Pietrzak, 2022). Research demonstrates that metacognitive training can enhance students’ task awareness and thinking skills, encourage translation strategy use, improve translation quality, and facilitate self-regulated learning (Fernández and Zabalbeascoa, 2012a, 2012b; Hu et al., 2021; Li and Yuan, 2022; Mellinger, 2019). For instance, Li and Yuan (2022) implemented a metacognitive-focused collaborative translation activity during the 17-week translation course, which effectively developed students’ metacognitive knowledge about person, task and strategies, promote their language awareness, deepen their autonomous and analytical thinking. Despite this, there remains a “relative dearth of scholarship on how metacognition can be incorporated into translator education programs or developed via assignments or tasks” (Mellinger, 2019, p. 607). Among the limited studies on metacognitive training in translation education (e.g., Fernández and Zabalbeascoa, 2012a, 2012b; Fernández and Arumi Ribas, 2014; Hu et al., 2021), the most commonly used training tools were metacognitive questionnaires which served as metacognitive prompts stimulating students to plan, monitor and evaluate their translation process. Metacognitive prompts can vary widely, from general questions to specific execution instructions, both aimed at enhancing students’ control over their information processing (Bannert and Mengelkamp, 2013). However, in previous studies, prompts were predominantly in the form of general questions. For example, in Fernández and Zabalbeascoa (2012a) study, prompting questions included “What translation problems do you think you have adequately solved and why?,” “Have you learnt anything new about the subject of the source text?,” etc. In contrast, specific prompts, which were found to be more effective for metacognitive training (Devolder et al., 2012), were used less frequently. Apart from that, the questionnaires in previous research typically consisted of a fixed set of items while failing to take into consideration task-specific features (e.g., text type and communicative function) which, however, can significantly influence students’ metacognitive processes (Dinsmore et al., 2015; Nilforoushan et al., 2023). In this view, there is a need for a metacognitive support tool that can offer specific and flexible prompts adaptable to various translation tasks. In this context, the metacognitive component of CSE translation scales emerges as a promising option as it includes a total of 89 metacognitive descriptors which are available for flexible selection based on the specific translation tasks. Appendix 1 presents a sample of these metacognitive translation descriptors. Evidently, these “can-do” descriptors are more specific than the general questions mentioned earlier and it is assumed that they can be useful tools for metacognitive training as has been demonstrated in previous studies related to speaking and writing skills (Glover, 2011; Mei et al., 2025).

### 2.2 Measurement of metacognitive strategy use

Given the central role of metacognitive strategies in metacognition (Reder and Schunn, 1996), many intervention studies are designed primarily to promote students’ use of metacognitive strategies (Bannert et al., 2009; Urban et al., 2023). To assess its potential improvements, both quantitative and qualitative methods have been employed. Unlike in general education where Likert-type metacognitive questionnaires have been the dominant measurement method, previous studies on metacognition in translation education (Hu et al., 2021; Li and Yuan, 2022; Mellinger, 2019) have largely relied on qualitative data, such as retrospective reports, observational notes, and interviews, presumably due to the absence of a rigorously developed and validated metacognitive questionnaire. In Li and Yuan (2022) study, for instance, students were asked to write a report reflecting on the entire process of metacognitive engagement during the translation activity and describing the strategies they used to address challenges. Evidently, the advantage of qualitative methods, compared to quantitative ones, is that they provide a more detailed picture of how students employ specific metacognitive strategies from a process-oriented perspective. Another thing worth noting is that a few studies (e.g., Hu et al., 2021) have employed Think-aloud Protocols (TAPs) to observe students’ metacognitive behaviors, however, the use of TAPs could unavoidably influence students’ translation process as has been long argued (Jakobsen, 2003). In light of this, it is imperative to incorporate non-disruptive process measures (e.g., screen recordings, retrospective reports) into research to gain a better understanding of metacognitive development.

### 2.3 The present study

As reviewed, metacognitive training has received limited attention in translation education, leaving the impact of metacognitive support on translation learners’ metacognitive development and performance largely unexplored. Moreover, it has been argued that standards-based metacognitive descriptors, compared to previous questionnaires (Fernández and Zabalbeascoa, 2012a, 2012b; Fernández and Arumi Ribas, 2014; Hu et al., 2021), can be a more advisable metacognitive training tool considering their greater specificity and flexibility (Glover, 2011; Mei et al., 2025). As such, this study aims to investigate how CSE metacognitive translation descriptors, as metacognitive support, affect students’ metacognitive strategy use and translation performance. The CSE was selected over the CEFR because it was specifically developed for the Chinese EFL context, making it more relevant to the study’s participants (i.e., Chinese EFL learners). The study is guided by three research questions:

RQ1. To what extent do CSE metacognitive translation descriptors affect students’ metacognitive strategy use in translation?

RQ2. To what extent do CSE metacognitive translation descriptors affect students’ translation performance?

RQ3. How do students perceive the usefulness of CSE metacognitive translation descriptors?

To address these questions, the study employs a pretest-posttest design, compares experimental and control groups, and incorporates both product- and process-oriented perspectives.

## 3 Methods

**Figure 1:**
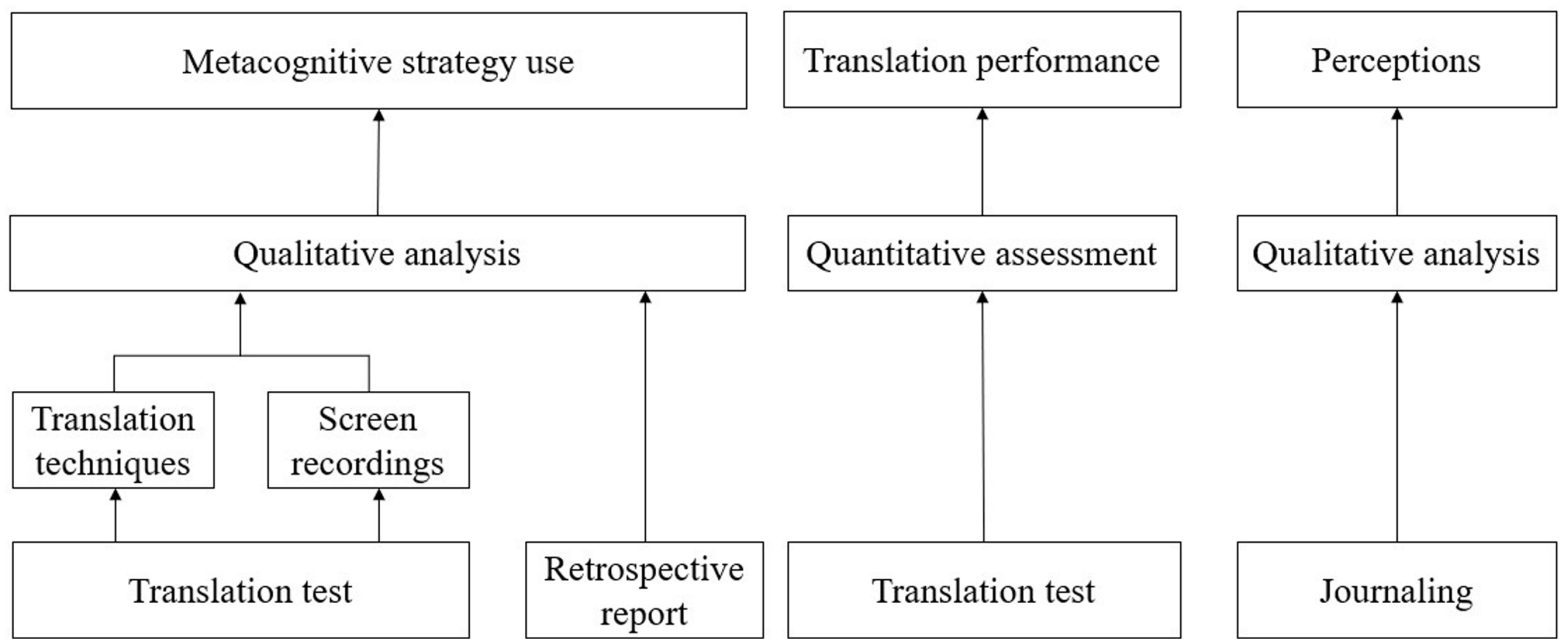
An overview of the research design.

To investigate students’ use of metacognitive strategies in translation (RQ1), both their translation products and processes were analyzed. For the products, translation techniques used in their translation outputs were examined. For the processes, metacognitive strategic behaviors observed in their screen recordings and retrospective reports were analyzed. To assess students’ translation performance (RQ2), a quantitative evaluation of their outputs was conducted. To explore students’ perceptions of the metacognitive support (RQ3), qualitative data collected from the experimental group’s journal entries were analyzed (see Figure 1 for an overview).

### 3.1 Participants

Using convenience sampling, a total of 40 third-year English major students who enrolled in the first author’s translation course participated in the present study. All of them agreed to participate and provided written informed consent. Before the course, the university had allocated them into two parallel classes, each consisting of 20 students. For the purpose of this study, one class was designated as the experimental group and the other as the control group. As the study focused on intervention within an authentic teaching environment, the use of convenience sampling was deemed appropriate due to the accessibility and availability of participants from the existing classes at the language institute (Creswell and Creswell, 2018; Han, 2024). Prior to the study, all students had passed the Test for English Majors Band 4 (TEM 4), a national English proficiency examination in China, roughly corresponding to CEFR B2 level. No significant differences were observed in their TEM 4 scores between the two groups (*p* = 0.986). Additionally, none of them had previous formal experience in attending a translation course and they had limited exposure to CSE translation descriptors. The primary distinction between the two groups was the presence or absence of metacognitive support embedded within the translation course.

### 3.2 Instruments

#### 3.2.1 Translation test

The study employed four parallel translation tasks for both pre- and post-tests, each consisting of two short English texts and two Chinese ones sourced from retired College English Test (CET) materials. Covering various genres (i.e., exposition, instruction, argumentation and description), the translation tasks in pre- and post-tests were confirmed with good content validity (Xu and Deng, 2023) and comparability of difficulty levels (Liu and Zheng, 2022). Tests were administered via computers at the beginning and end of the 16-week semester, with each session lasting about 50 min. At the start of each test session, students were instructed to activate the screen recorder on their computers, ensuring that their entire translation process was documented through screen recordings.

#### 3.2.2 Retrospective report

Retrospective report, when overlaid with screen recordings, can provide more powerful evidence about students’ translation process (Saldanha and O’Brien, 2013). Retrospective, rather than concurrent report, was used because the latter has a slow-down effect on students’ translation process and could then affect their translation performance (Jakobsen, 2003). In the present study, retrospective reports were conducted in both groups immediately after the pre- and post-translation tests to examine students’ metacognitive strategy use throughout the translation process, providing supplementary evidence for the screen recording results and thus capturing a more comprehensive picture of students’ metacognitive behaviors. Specifically, students were asked to recall their translation process and verbalize any strategic behaviors or activities they had employed during the task.

#### 3.2.3 Journaling

Journaling and interviews have long been used to investigate students’ perceptions of certain pedagogical interventions (Chen et al., 2023; Glover, 2011; Han, 2024; Li and Yuan, 2022). In this study, journaling was used in lieu of interviews as the former “allowed for complete integration into coursework separate from sequential thinking and thought examination from a holistic perspective” (Teng, 2020, p. 558). At the end of the semester, students in the experimental group were asked to complete a short journal. Specifically, they were instructed to write down their perceptions of and experiences with the metacognitive support. Drawing on question designs from previous studies mentioned above, the guiding questions for the journal entries included: “What do you think about the usefulness of the metacognitive descriptors?,” “Have the metacognitive descriptors influenced your translation learning?,” and “Did you encounter any difficulties in using the metacognitive descriptors?”

### 3.3 Research settings and metacognitive intervention

The study was conducted in a general Chinese-English (C-E)/English-Chinese (E-C) translation course at a key university in China. The 16-week course included weekly 90-min sessions. During the first 4 weeks, the basics of translation (e.g., translation theories, strategies, methods, and techniques) were taught. During this period, the instructor implemented direct metacognitive training before transitioning to indirect training. As previously reviewed, direct training involves explaining metacognitive knowledge and strategies while demonstrating their application (Bannert and Mengelkamp, 2013). Accordingly, the instructor first delivered a lecture explicitly explaining the metacognitive fundamentals (e.g., definition, types, importance) and the CSE metacognitive translation descriptors. Subsequently, the instructor demonstrated the application of these descriptors through an in-class think-aloud activity which helped students understand when and how to use these metacognitive components (Hartman, 2001). For the remaining 12 weeks, students were assigned 12 translation tasks, evenly split between E-C and C-E translations. Throughout these weeks, indirect training measures (i.e., metacognitive support in the form of CSE metacognitive translation descriptors) were integrated into the course. The course in both groups followed a learner-centered pedagogical approach (Li, 2017), consisting of the following procedures: (1) Individual translation: students translated assigned texts independently before class; (2) Group discussion: students then discussed in groups to jointly produce a final translated text; (3) Classroom presentation: one group presented their translation in class, and other groups provided comments; (4) Individual reflection: students completed a written reflection after class.

**Figure 2:**
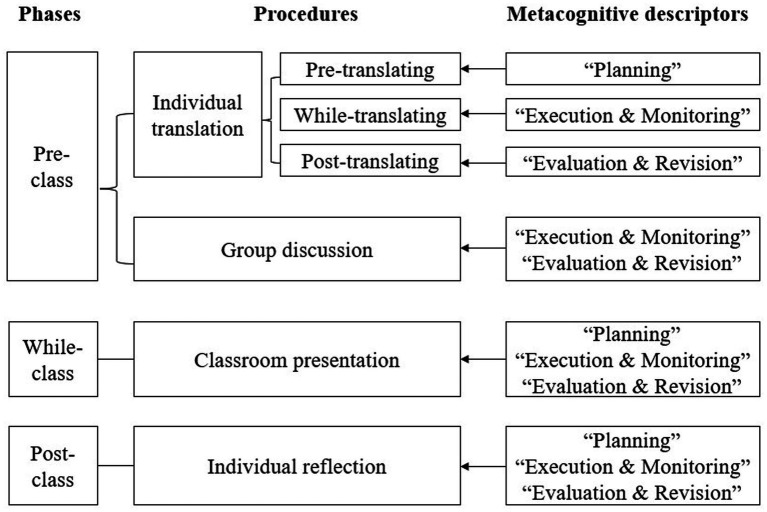
An overview of the metacognitive intervention.

Drawing on Pietrzak (2022) proposed model of metacognitive support which incorporates metacognitive activities into the three phases of translation practice (i.e., pre-practice, in-practice, post-practice), the present study integrated metacognitive descriptors into different phases of the translation course for the experimental group. Since students’ metacognitive strategy use might be affected by task characteristics (Dinsmore et al., 2015; Nilforoushan et al., 2023), the descriptors used in each session were not identical; rather, they were selected from the CSE metacognitive translation descriptor pool (89 descriptors) based on the specific content of the assigned translation tasks. The following section provides details on how these descriptors were employed as metacognitive support (see Figure 2 for an overview).

As illustrated, before engaging with the individual translation task, students in the experimental group were required to carefully read the descriptors listed in the “planning” section. During the translation process, they were required to refer to the descriptors in the “execution & monitoring” section and integrate them into their translation work. After completing their initial draft, students evaluated and revised their translations using the descriptors in the “evaluation & revision” section. During group discussions, they were reminded to consult the “execution & monitoring” and “evaluation & revision” descriptors. The teacher supported this metacognitive process by explicitly mentioning relevant descriptors while providing feedback during classroom presentations. After class, when writing reflections on their translation processes and products, students were instructed to review all the descriptors thoroughly and conduct a self-assessment by scoring each descriptor (1 = strongly disagree, 7 = strongly agree). To ensure consistent attention to the use of descriptors throughout the learning process, students were encouraged to take necessary notes using the provided descriptor checklist (see Appendix 1 for a sample of descriptor checklist).

### 3.4 Data analysis

For the quantitative analysis, the two groups were compared based on their pre- and post-test translation scores. Students’ translations were assessed using the CET translation rating scale (National College English Testing Committee, 2016; see Appendix 2), a 15-point holistic rubric with five bands. Two experienced translation teachers, each with over 5 years of CET translation scoring experience, independently rated the translations. When discrepancies exceeded 3 points (one band), a third rater with the same qualifications was consulted. Inter-rater reliability was high, with correlation coefficients of 0.909 for the pre-test and 0.926 for the post-test. The average score from the two raters served as the quantitative measure of each student’s translation performance. To examine the intervention’s effects, linear mixed-effects models (LMMs) were conducted in SPSS (version 27.0), with time (pre-test vs. post-test) as a within-subject variable and group (experimental vs. control) as a between-subject variable. Test scores were the dependent variable. LMMs were chosen for their suitability in analyzing small sample sizes and accounting for variance across both items and individuals (Wiley and Rapp, 2019). A preliminary assessment confirmed that the model met necessary assumptions, including normality, linearity, and homogeneity of variance. The *p*-value for statistical significance was set at 0.05 and effect size was reported using Cohen’s *d*.

For the qualitative analysis, both textual analysis and thematic analysis were conducted using NVivo 11. Textual analysis was employed to examine translation techniques in students’ translation outputs, while inductive thematic analysis was applied to process-oriented data derived from screen recordings and retrospective reports. Textual analysis is a method that “closely examines either the content and meaning of texts or their structure and discourse” (Given, 2008, p. 865). In the case of translation, textual analysis can help identify the translation techniques used in the translated text through a detailed examination of its content, meaning and structure. In this study, Xiong (2014) classification of translation techniques (i.e., addition, omission, division, combination and conversion) served as a framework for categorizing the strategies employed by students in their translations. Thematic analysis, which aims at “identifying, analyzing and reporting patterns (themes) within data” (Braun and Clarke, 2006, p. 79), was used in this study as it helped identify specific metacognitive behaviors as potential themes from the screen recordings and retrospective reports. For example, if a student was observed or reported to have searched relevant information related to the translation tasks, this behavior could be coded as “search for background information.” It should be noted that each translation technique or strategic behavior was calculated only once per student, as this method is sufficient to evaluate whether and how students could use certain metacognitive strategies in their translation process. More importantly, it is sometimes difficult to quantify the frequency of certain behaviors (e.g., revising a sentence back and forth) and it is also unreasonable to conclude that repeating the same behavior (e.g., searching words in the dictionaries) would always indicate a higher level of metacognitive strategy use (Mei et al., 2025). Given the analysis of metacognitive strategies involved nominal coding (e.g., presence/absence of metacognitive behaviors), Cohen (1960) Kappa was used for assessing inter-coder reliability. The kappa value of 0.84 indicated strong agreement (Landis and Koch, 1977). To explore students’ perceptions of metacognitive support, inductive thematic analysis was also employed. Data relevant to students’ perceptions were identified and coded. Two independent coders classified the data and resolved any discrepancies through discussion.

## 4 Results

### 4.1 Metacognitive strategy use

#### 4.1.1 Strategy use from product-oriented perspectives

Table 1 presents the number of students using various translation techniques in both the experimental (E) and control (C) groups during the pre- and post-translation tests. As shown, the number of students using each technique was fairly similar in the pre-test with differences of no more than two. However, these differences increased in the post-test. Chi-squared test revealed no significant differences between the two groups in the pre-test for any of the translation techniques: addition (*p* = 0.723), omission (*p* = 0.633), division (*p* = 0.490), combination (*p* = 0.519), and conversion (*p* = 0.525). In the post-test, however, significant differences were found in the use of the “addition” (*p* = 0.047) and “division” (*p* = 0.025), while no significant differences were observed for the other three techniques: omission (*p* = 0.337), combination (*p* = 0.736), and conversion (*p* = 0.114).

**Table 1:** Number of students using translation techniques in the pre- and post-test.

	E (pre)	C (pre)	E (post)	C (post)
Addition	6	5	16	10
Omission	2	3	10	7
Division	7	5	15	8
Combination	7	9	14	13
Conversion	8	10	18	14

#### 4.1.2 Strategy use from process-oriented perspectives

Table 2 presents the codes identified from the screen recordings and retrospective reports of students’ translation processes, revealing their metacognitive strategic behaviors during the translation tests. As shown, a total of 16 types of strategic behaviors were identified, with seven categorized under “planning” and nine under “revision.” Similar to the findings regarding the students’ use of translation techniques, both groups were found to be comparable in the pre-test, with Chi-squared tests revealing no significant differences in any strategic behavior (*p* > 0.05). However, in the post-test, Chi-squared tests identified significant differences in seven strategic behaviors: “analyzing the style” (*p* = 0.047), “analyzing the readership and purpose” (*p* = 0.027), “consulting parallel texts” (*p* = 0.008), “revising capitalizations and punctuations” (*p* = 0.028), “revising mistranslations” (*p* = 0.011), “revising incomplete information” (*p* = 0.027) and “revising inconsistencies” (*p* = 0.038).

**Table 2:** Students’ metacognitive strategic behaviors in the pre- and post-test.

Theme	Code	E (pre)	C (pre)	E (post)	C (post)
Planning	Looking up words in online dictionaries	17	18	20	20
	Analyzing the style	6	4	16	10
	Analyzing the textual structure	5	6	15	14
	Searching for background information	3	3	15	13
	Analyzing text types and genres	3	4	14	10
	Analyzing the readership and purpose	0	0	13	6
	Consulting parallel texts	0	0	6	0
Revision	Revising grammatical mistakes	13	15	20	19
	Revising capitalizations and punctuations	9	8	18	12
	Revising sentence structures	5	6	15	10
	Revising the logic	5	5	14	9
	Revising mistranslations	4	3	14	6
	Revising incomplete information	3	2	13	6
	Revising collocations	3	5	12	8
	Revising repetitions with synonyms	0	1	10	5
	Revising inconsistencies	0	0	9	3

### 4.2 Translation performance

Table 3 displays the descriptive statistics of students’ total scores in the translation tests. LMM analysis did not reveal significant interaction effect between time and group (*b* = 2.18, *SE* = 1.38, *t* = 1.58, *p* = 0.119, Cohen’s *d* = 0.63), suggesting that the metacognitive support did not pose a statistically observable impact on students’ overall translation performance. The main effect of time was highly significant (*b* = 2.45, *SE* = 0.98, *t* = 2.51, *p* < 0.001), indicating that performance significantly improved from pre-test to post-test across participants in both groups. However, the main effect of group was not significant (*p* = 0.128), suggesting that, regardless of time, there was no overall difference between the experimental and control groups.

**Table 3:** Descriptive statistics of students’ total translation scores.

	N	Min.	Max.	M	SD
Pre-test (experimental)	20	35.00	45.00	41.18	2.74
Pre-test (control)	20	31.00	44.50	41.20	3.01
Post-test (experimental)	20	38.50	52.50	45.80	3.37
Post-test (control)	20	34.50	50.00	43.65	3.48

In light of this, a further examination into their E-C/C-E translation scores was conducted (see Table 4). LMM analysis revealed a significant interaction effect between time and group in E-C scores (*b* = 1.75, *SE* = 0.80, *t* = 2.20, *p* = 0.031, Cohen’s *d* = 0.96), indicating a significant intervention effect on E-C performance. However, no intervention effect was found for C-E performance as the interaction effect between time and group was not statistically significant (*b* = 0.43, *SE* = 0.80, *t* = 0.53, *p* = 0.598, Cohen’s *d* = 0.17).

**Table 4:** Descriptive statistics of students’ E-C/C-E translation scores.

	E-C				C-E			
	Min.	Max.	M	SD	Min.	Max.	M	SD
Pre-test (experimental)	18.00	23.00	20.43	1.36	16.00	23.50	20.75	1.77
Pre-test (control)	14.00	23.50	20.35	2.06	17.00	24.00	20.85	1.62
Post-test (experimental)	20.50	27.00	23.15	1.86	17.00	25.50	22.65	1.89
Post-test (control)	16.00	24.00	21.33	1.94	18.50	27.00	22.33	2.05

### 4.3 Students’ perceptions

Thematic analysis of students’ journal entries resulted in eight major codes, which were categorized into two themes (see Table 5). As shown, the CSE metacognitive translation descriptors were reported to have a positive influence on both students’ translation process and translation learning. Regarding the former, students mentioned that the descriptors facilitated their pre-translation preparation and post-translation reflection. Additionally, they believed the descriptors provided valuable guidance for revising their translations and helped them decide which translation version to choose. Regarding the latter, students viewed the descriptors as a diagnostic tool that highlighted their translation strengths and weaknesses. They also appreciated the opportunity for cooperative learning enabled by the descriptors. Furthermore, they emphasized that the self-assessment section in the descriptor checklist allowed them to visualize their progress over time. Finally, they reported enhanced strategic awareness through descriptor use.

**Table 5:** Coding results of students’ perceptions.

Theme	Code	Data (Example)
Translation process	Facilitating pre-translation preparation	“Descriptors such as ‘can build up term bases in relevant fields using commonly used translation software’ helped me thoroughly prepare for translating political documents, making the task feel less daunting.” (student 3)
	Facilitating post-translation reflection	“The descriptors made my post-translation reflection more focused and specific, guiding me to concentrate on aspects where I underperformed.” (student 15)
	Offering clues for revision	“When I’m unsure about how to revise, the descriptors served as an important reference indicating key points I overlooked.” (student 5)
	Providing rationale for translation decision	“When I have multiple translation versions, the descriptors helped me decide which to choose by providing clear rationale and criteria, making the choice more convincing.” (student 16)
Translation learning	Conveying diagnostic feedback	“The checklist helped me recognize my strengths and weaknesses in translation while enabling me to set targeted goals and plans for improvement.” (student 10)
	Promoting cooperative learning	“Some descriptors in the checklist encouraged me to seek feedback and guidance from classmates, fostering cooperative learning.” (student 9)
	Visualizing progress overtime	“The checklist helped me track my progress in translation. As my self-assessment scores increased, my improvements became tangible.” (student 17)
	Increasing strategic awareness	“In the post-test, I clearly noticed my translation had improved. With the descriptors, I consciously applied strategies like adjusting word choices and modifying sentence structures. I no longer translated as casually as before.” (student 1)

## 5 Discussion

This study has explored the role of CSE metacognitive translation descriptors as metacognitive support in promoting translation learners’ metacognitive strategy use and translation performance. Results indicated that the descriptors could evidently improve translation learners’ metacognitive strategy use, corroborating the effectiveness of metacognitive support on metacognitive development (e.g., Bannert et al., 2009; Künsting et al., 2013; Urban et al., 2023; Zumbach et al., 2020). However, contrary to previous findings (Fernández and Zabalbeascoa, 2012b; Hu et al., 2021), no statistical improvement has been found in students’ overall translation performance.

On the one hand, product-oriented analysis of students’ use of translation techniques indicated that the experimental group outperformed the control group in executing certain metacognitive strategies (e.g., addition, division) during translation. This finding aligns with Fernández and Zabalbeascoa (2012a) study which observed enhanced awareness of translation strategies (e.g., adding grammatical subjects, adding information implicit in the source text) after the metacognitive translator training. On the other hand, process-oriented analysis of students’ metacognitive behaviors revealed that those receiving metacognitive support were significantly more effective in orchestrating some strategies related to planning and revision. While previous studies primarily examined perceived strategy use through self-reported data (Fernández and Arumi Ribas, 2014; Glover, 2011; Mellinger, 2019; Teng, 2020), the current study provides empirical evidence through direct analysis of students’ actual strategy application, yielding a more comprehensive understanding of students’ metacognitive behaviors. Consistent with Hu et al. (2021) findings of increased metacognitive strategy use (i.e., planning, monitoring, evaluation) in the experimental group, this study has further delineated specific strategies within each dimension, offering more nuanced insights into students’ metacognitive processes.

Noticeable improvements were observed in overall translation performance across participants in both groups overtime, supporting the effectiveness of Li (2017) pedagogical model in enhancing translation competence. That being said, inter-group comparison showed no statistical impact of metacognitive support on students’ overall translation performance. This may be attributed to the relatively short duration of the training time, that is, despite the 16-week course period, translation practice sessions were limited to 12 weeks. Consequently, the experimental group might not have fully internalized the descriptors, potentially impeding their ability to effectively activate metacognition to enhance their translation performance. This points to the importance of sufficient training time as suggested by previous literature on metacognitive training principles (Bannert, 2006; Bannert and Mengelkamp, 2013; Veenman et al., 2006). It is anticipated that the intervention’s effect would become significant if the metacognitive training were implemented over an extended period (e.g., 16 training weeks or longer) as demonstrated in previous studies (Li and Yuan, 2022; Mei et al., 2025). However, it is also possible that an inverse relationship between metacognitive strategy use and translation performance would emerge after students develop greater translation expertise. This is because previous findings have shown that experienced translators would exhibit greater automaticity and reduced metacognition in translation tasks (Shreve, 2009).

Further analysis of the E-C/C-E translation scores revealed an interesting pattern: metacognitive support notably influenced students’ E-C translation performance, implying that metacognition might play a more significant role in E-C translation competence compared to the C-E one. One possible explanation could be that the target language in E-C translation is students’ native language (i.e., Chinese) which has reached maturity, thus making non-linguistic factors such as metacognition more influential. Conversely, in C-E translation, the target language is their foreign language (i.e., English) which is still undergoing development, linguistic factors are likely to retain greater prominence. This finding is worth further investigation as previous studies (e.g., PACTE, 2005; Yang and Li, 2021) on translation competence have predominantly focused on identifying its constituents/sub-competences while paying much less attention to how the relative importance of certain constituent (e.g., metacognitive strategic competence) could be affected by translation directions. Discoveries in this respect could inform the priority order among different constituents in teaching direct translation (i.e., into the native language) and inverse translation (i.e., into the foreign language), which would provide valuable insights into translator training.

Qualitative analysis of students’ journal entries revealed their positive experiences with the metacognitive support. The responses emphasized the beneficial role of the descriptors in making students’ translation process more organized by guiding them through the “pre-translating planning--while-translating execution/monitoring--post-translating evaluation/revision” cycle. Additionally, it is found that the descriptors have made students’ translation process more focused by directing their attention to the most important aspects during each phase. This finding can be attributed to the specificity of the descriptors which, compared to general prompts, tend to be more effective in guiding students’ attention toward the essential components of the task or the specific features of a problem (Devolder et al., 2012). In this context, the instructor’s role in selecting appropriate descriptors for each translation assignment becomes rather crucial as effective descriptor selection can guide students effectively, whereas poor selection may mislead them. This collaborates the argument that teachers have a critical role to play in metacognitive training (Lee and Mak, 2018). Beyond influencing the translation process, the metacognitive support also made students’ learning more targeted (by providing diagnostic feedback), observable (by visualizing progress over time), and strategic (by increasing strategic awareness), which empowered students to become agents of their own learning actively engaged in setting personalized learning goals, monitoring learning progress and enacting learning strategies (Braad et al., 2022; Echeverri, 2015). These findings resonate with Li and Yuan (2022) study which demonstrated that metacognitive training benefited not only students’ translation process but also their long-term autonomous and self-regulated learning development. Another noteworthy benefit highlighted by students was the positive role of the descriptors in fostering cooperative learning. This can be attributed to the inclusion of relevant metacognitive descriptors in the CSE, which encouraged interactive activities such as consulting professionals (“can consult relevant professionals to ensure comprehension of the professional knowledge in the original”) and seeking peer feedback (“can correct any mistranslations of main ideas through cross-checking”). These collaborative activities, stimulated by the CSE descriptors, not only enriched students’ learning experiences but also served as a significant impetus for promoting students’ metacognitive development, as demonstrated in previous research (De Backer et al., 2015; Kim and Lim, 2018). Compared with Mei et al. (2025) study that used CSE writing descriptors for metacognitive training without collecting students’ feedback on the intervention, the present study gathered and analyzed students’ responses, thereby providing additional evidence of metacognitive training’s effectiveness from learners’ perspectives.

## 6 Conclusion

Motivated by the limited attention given to metacognitive training in translation education and its demonstrated benefits for students’ translation learning, this study implemented standards-based translation descriptors as a metacognitive training tool in a translation course with an aim to examine the effectiveness of CSE descriptors in enhancing learners’ metacognitive development and translation performance. Overall, this study yielded positive results of the metacognitive intervention on students’ metacognitive strategy use and translation learning. Theoretically, this study can help establish a metacognitive translator training model by systematically embedding metacognitive prompts into the three phases of translation (i.e., pre-translating, while-translating and post-translating). As previous metacognitive training research has predominantly focused on general language education (e.g., listening, writing), this study enriches the existing literature by extending metacognitive research to translation which is an essential component of language curricula. Practically, by incorporating metacognitive components of established language standards (e.g., CSE) into Li (2017) pedagogical model, this study can provide important implications for implementing standards-based descriptors in regular translation activities including individual translation, group discussion, classroom presentation and individual reflection. The integration of metacognitive support into routine learning activities is expected to benefit students’ self-regulated learning in the long run. As one of the first attempts to apply CSE metacognitive descriptors for instructional purposes, this study can also provide a new approach to metacognitive instruction in language education. For example, language teachers can use the metacognitive components of CSE listening and speaking scales to promote students’ metacognitive knowledge and strategy use in those skills.

In spite of the contributions, some limitations exist. Firstly, as with many other classroom-based metacognitive intervention studies (Chen et al., 2023; Han, 2024), the sample size in this study is quite small. For future studies, it is recommended to include larger sample sizes that encompass a broader range of translation proficiency levels, thereby providing a more comprehensive understanding of the impact of metacognitive support and improving the generalizability of the research findings. Secondly, this study relies solely on qualitative measures of metacognitive strategy use due to the absence of validated assessment tools. This highlights the need for future work to develop and validate a metacognitive translation strategies inventory, which would provide more robust evidence of students’ metacognitive development.

## Data Availability

The raw data supporting the conclusions of this article will be made available by the authors, without undue reservation.
